# A Multi-Stage Dual Encoder–Decoder Network Based on Event Image Cross-Modal Fusion for Image Deblurring

**DOI:** 10.3390/s26154762

**Published:** 2026-07-27

**Authors:** Yan Liu, Yanfei Jia, Sheng Qiang, Yongpei Lin, Liquan Zhao

**Affiliations:** 1 College of Electrical and Information Engineering, Beihua University, Jilin 132021, China; 2 State Grid Jilin Information & Telecommunication Company, Changchun 130022, China; 3 College of Electrical Engineering, Northeast Electric Power University, Jilin 132021, China

**Keywords:** image deblurring, event image, deep convolutional network, deep learning

## Abstract

Most existing event-driven image deblurring methods ignore inherent differences between the two modalities and lack explicit alignment strategies, leading to cross-modal mismatches and degraded feature reconstruction. To address this issue, a multi-stage dual encoder–decoder image deblurring method based on event image cross-modal fusion is proposed. The proposed network consists of an encoder and a decoder. The encoder employs dilated convolutional residual modules for feature extraction. It also integrates a cross-modal feature fusion module and a local scoring mechanism. These components combine event features with frame image features while suppressing noise. The decoder reconstructs image features via two directional decoding sub-networks. It also incorporates a feedback attention module. This module selects informative features along the feedback path. As a result, the image reconstruction quality is enhanced. In addition to the standard loss, mean absolute error, structural similarity, and frequency reconstruction losses are used to optimize deblurring performance. Extensive experiments are conducted on the GoPro, REBlur, and RwEvent datasets. For PSNR, our method exceeds REFID by 0.23 dB, 0.20 dB, and 0.49 dB on the three datasets. For SSIM, our model achieves gains of 0.002, 0.002, and 0.017 against REFID. In terms of computational cost and inference speed, our network adds only 3.4 M parameters and 105.2 GFLOPs, with an FPS reduction of only 2.12. This trivial efficiency loss delivers significant improvements in both pixel and structural restoration performance. Ablation experiments verify the independent positive contribution of each designed module. Both qualitative visual comparisons and quantitative metrics demonstrate that the proposed network has stronger deblurring and generalization capabilities.

## 1. Introduction

With the rapid advancement of image sensing and processing technologies, image-based applications have become increasingly prevalent and important. These applications include image classification, object detection, and semantic segmentation. However, the performance of these applications heavily depends on the quality of the input images [1].

Image blur is a common degradation that adversely affects image quality. It can arise from various sources. These include motion blur caused by fast camera or object movement. They also include optical blur due to defocus or lens contamination. Another source is blur from long exposure times in low-light environments. The goal of image deblurring is to restore a sharp image by recovering lost details. This improves both the visual quality of the image and its performance in downstream vision tasks.

In recent years, deep learning has developed rapidly. Remarkable progress has been achieved in a wide range of computer vision tasks [2]. Consequently, numerous deep learning-based image deblurring algorithms have been proposed. To more effectively address the deblurring problem, modern deep learning approaches typically train a convolutional neural network (CNN) on large-scale datasets. These networks learn the implicit mapping between blurred and sharp images [3,4]. Although these methods achieve impressive performance on existing public datasets, they often fail when confronted with severe motion blur or real-world complex scenes. Their effectiveness depends heavily on the type and diversity of blur in the training data. To enhance the modeling capability for rapid dynamic changes, recent studies have introduced event cameras as an auxiliary source of information. Event cameras offer high temporal resolution. They are inherently free from motion blur. This provides neural networks with more precise dynamic cues. As a result, the model’s generalization ability and restoration quality are improved under challenging dynamic blur conditions.

Event cameras are bio-inspired asynchronous sensors [5,6]. They offer microsecond-level temporal resolution. They also perform exceptionally well in high-dynamic-range environments. As a novel class of neuromorphic sensors, event cameras capture visual information in a fundamentally different manner compared to conventional frame-based cameras. Instead of recording full image frames at fixed intervals, event cameras independently detect brightness changes at each pixel. This detection occurs whenever the change exceeds a predefined threshold. The resulting data is encoded as a stream of events. These events represent the intensity changes occurring during the exposure time of a conventional frame. This unique mechanism enables the development of event-based image deblurring methods [7].

With advances in event camera technology, event-based motion deblurring methods have been proposed. These methods address the loss of motion information that occurs during the imaging process of frame-based cameras. Event cameras asynchronously output event data with low latency. They are inherently immune to motion blur, even at high speeds. Moreover, they can continuously capture texture and motion information over time. Event-based deblurring has demonstrated promising results in the field. However, existing methods typically fuse features from the two modalities using simple convolution or attention mechanisms. These methods lack explicit modeling of the deep collaborative relationships between image and event modalities. The fusion strategies and reconstruction framework design thus remain to be further improved.

To address these issues, this paper proposes a dual-decoder image deblurring method based on event image cross-modal fusion. The main contributions of this work are as follows:

1. A dual-stage cross-modal fusion paradigm consisting of Cross-Modal Feature Fusion Module (CFFM) and Local Score-based Feature Fusion Module (LSFF) is designed. Unlike previous methods that directly concatenate event and image features without alignment, CFFM uses bidirectional multi-head attention to eliminate inter-modal distribution discrepancies, while LSFF calculates local feature-matching scores to filter out irrelevant event noise, jointly achieving accurate denoising fusion of bimodal information.
2. A rotation-sharing dual-decoder reconstruction architecture is proposed. Unlike conventional single-decoder networks that adopt undifferentiated reconstruction for all blur directions, two decoders share base convolution kernels with spatial rotation transformation to independently model horizontal and vertical motion blur, mitigating structural distortion caused by complex multi-orientation non-uniform blur.
3. A hierarchical Feedback Attention Module (FAM) is embedded into the encoder–decoder pipeline. High-level semantic features are fed back to shallow layers as attention weights to dynamically screen for discriminative texture features, compensating for detail loss in severely blurred regions and further elevating the sharpness of reconstructed images.

The rest of this paper is structured as follows. Section 2 reviews related work on image deblurring and event-based vision restoration. Section 3 provides details of our proposed event-driven image deblurring method. Section 4 presents the experimental simulations and a comprehensive discussion of the results. Finally, Section 5 concludes this paper and summarizes the overall findings.

## 2. Related Works

The clear reconstruction of motion-blurred images has long been a key research topic in computer vision [8,9]. Traditional deblurring methods primarily rely on kernel estimation and regularization. These approaches include deconvolution and kernel estimation [10]. This type of method does not require large training datasets and has clear mathematical interpretability. However, its performance degrades sharply when faced with complex, non-uniform motion blur in real scenes. Hand-crafted priors cannot adapt to diverse real-world blur distributions, leading to severe artifacts in restored images and greatly limiting their practical value.

In recent years, deep learning has achieved great success. CNN-based methods have made significant breakthroughs in deblurring performance. These methods directly map blurred images to their latent sharp counterparts. Sun et al. [11] combined CNN patch regression with traditional energy optimization to predict blur kernels. This method improves the stability of kernel estimation for simple uniform blur, yet it still relies on manually designed energy terms and fails for irregular non-uniform motion blur. Fang et al. [12] proposed a self-supervised method for non-uniform blur kernel estimation. This method is based on optical flow motion priors. It models the distribution of blur kernels as a latent variable using a regularized flow. Uncertainty modeling is also introduced to improve the stability and robustness of kernel estimation.

Li et al. [13] employed a similar degradation encoder for the same purpose. Their method can adapt to various blurs without relying on hand-crafted kernels or priors. Multi-stage networks have demonstrated impressive performance in advanced vision tasks. Nevertheless, the network only takes RGB images as input and lacks access to microsecond-level temporal motion information, so it cannot recover fine textures under extreme motion blur. Inspired by this success, multi-stage designs have also been introduced into image deblurring. Kupyn et al. [14] regarded image deblurring as a special case of image-to-image translation. This was inspired by image super-resolution and image-to-image translation in Generative Adversarial Networks (GANs). They proposed a GAN-based deblurring network. The generator and discriminator are trained alternately to enhance deblurring performance. Perceptual loss and adversarial loss are used as the overall objective. The generator can recover rich visual details for moderate blur, but GAN training is prone to mode collapse, and the model lacks an explicit multi-scale feature extraction ability for complex dynamic scenes. Nah et al. [15] introduced a deep multi-scale convolutional neural network. This network restores blurred images in an end-to-end manner. It achieved excellent performance in dynamic scene deblurring. However, this method uses only single-blurred RGB frames as input and cannot obtain ultra-high-temporal-resolution motion records provided by event cameras. When handling scenes with ultra-fast, non-uniform motion, the network cannot capture transient brightness changes, leading to severe edge blurring and the loss of fine textures on rapidly moving targets. Tao et al. [16] proposed a multi-scale recurrent network. This network leverages weight sharing to simplify the network structure. It also reduces the number of parameters and facilitates easier training. Similar to Ref. [15], this recurrent architecture relies solely on frame images and has no access to asynchronous event motion cues, so it still cannot overcome the restoration bottleneck under severe high-speed motion blur.

Although these deep networks demonstrate notable advantages in image deblurring, they rely solely on the imaging modality. They cannot model instantaneous dynamics. As a result, they struggle to capture blur caused by high-speed motion explicitly. This leads to limitations in inter-frame motion perception when handling severe motion scenarios.

In recent years, research on utilizing event information to assist deblurring tasks has become increasingly prominent. Event cameras are bio-inspired sensors. They can asynchronously capture intensity changes at each pixel with high temporal resolution. They also provide low-latency visual information. Their high temporal resolution and low latency enable accurate edge detection. They also encode rich motion timing information within the event stream. This information is crucial for reconstructing latent sharp images. Event-driven motion deblurring aims to fuse the complementary characteristics of events and images. The goal is to achieve high-quality image restoration. Mueggler et al. [17] adopted a gain-adaptive complementary filter to fuse frames and events for static scene reconstruction. This hand-designed filtering mechanism has high inference speed, but it is a non-learning method and cannot adaptively fuse multi-modal features for complex dynamic scenes. Liu et al. [18] innovatively integrated spiking neural networks with convolutional neural network. A motion-guided mechanism was proposed. This mechanism fully exploits the temporal characteristics of event cameras. It achieves efficient and accurate dynamic scene deblurring. This method not only improves deblurring performance but also offers new insights into neural network design for event-driven vision tasks.

Jian et al. [19] began with a sequence-based formulation of event-based motion deblurring. An end-to-end convolutional recurrent neural network was proposed. The global–local fusion strategy improves feature utilization, but the network fails to account for the statistical inconsistency in bimodal feature spaces, leading to cross-modal mismatch during feature fusion. To further enhance reconstruction results, a differentiable directional event filtering module was introduced. This module extracts rich boundary priors from the event stream. Chen et al. [20] proposed a residual learning-based method for image deblurring and high-frame-rate video reconstruction. This method fully leverages the high temporal resolution advantage of event cameras in dynamic scenes. Blurred images and event streams are taken as inputs. A unified neural network architecture is constructed. It restores images by predicting the residual between blurred and sharp images. It also simultaneously estimates inter-frame motion information from the event stream. This generates continuous high-frame-rate sharp video sequences. However, the feature fusion module uses only basic convolutional layers without explicit cross-modal attention alignment, so noise in event streams can interfere with frame feature reconstruction. Wang et al. [21] were the first to propose a joint task of image deblurring and image super-resolution based on a sparse coding framework. A cascaded scheme of deblurring and super-resolution networks was introduced. This multitask design improves feature reuse efficiency, but the sparse coding module introduces significant computational overhead and lacks real-time inference capability. Asymmetric supervision was employed to effectively perform upsampling of event data. Zhang et al. [22] exploited the uniqueness of adjacent frames in video sequences. A supervision strategy from blurred to sharp images was proposed. Sharp frames beyond the exposure time are recovered. This unified event-camera-based deblurring and event-camera-based video frame interpolation tasks. The uniqueness of sharp frames corresponding to blurred images with different exposure times was considered. Still, the fusion scheme treats event and frame features equally without screening effective motion-related event signals, leaving background noise to degrade restoration effects. Geng et al. [23] proposed an event-enhanced deblurring framework tailored for snapshot mosaic hyperspectral cameras. An Event-enhanced Hyperspectral frame Deblurring Network with Mosaic-aware Spatial–Spectral Attention and Event-Frame Mutual Gating modules is developed to achieve effective cross-modal spatial–spectral feature fusion. However, the method relies on explicit spectral calibration and lacks explicit cross-modal spatial attention alignment, which may limit its generalizability to conventional RGB deblurring tasks. Weng et al. [24] proposed BVSR-EvD, a diffusion-based framework that leverages events to simultaneously perform blurry video deblurring, spatial super-resolution, and temporal interpolation. A Trident Diffusion Model is designed to decouple and dynamically recombine three data priors—motion prior from events, content prior from videos, and physical prior from their integration—enabling up to 8× spatial and 64× temporal resolution enhancement. However, the dynamic prior integration relies on learned weight maps that may not generalize to unseen motion patterns, and the framework’s effectiveness is highly dependent on the temporal alignment quality between sparse events and video frames. Fu et al. [25] proposed EDVD, a cross-modal spatio-temporal fusion method for video deblurring. A cross-modal collaborative attention mechanism is designed to fuse features from blurred frames and event frames, thereby deeply extracting motion information from event frames. This method achieves state-of-the-art performance on both synthetic and real datasets. However, the diffusion prior and event features are fused in a sequential rather than synergistic manner, so the interaction between the two modalities remains insufficient for severely blurred regions.

Although event-camera-based deblurring addresses the issues faced by traditional methods, some challenges remain. These issues include loss of motion information and severe blur recovery in dynamic scenes. Most existing approaches still face challenges, such as insufficient inter-modal information fusion and limited feature reconstruction capabilities. To better exploit correlations between event and image features and improve the restoration quality of blurred regions, this paper proposes a dual-decoder image deblurring method based on event image cross-modal fusion.

## 3. Proposed Image Deblurring Method Based on Event-Driven

### 3.1. Complete Network Architecture

**Figure 1:**
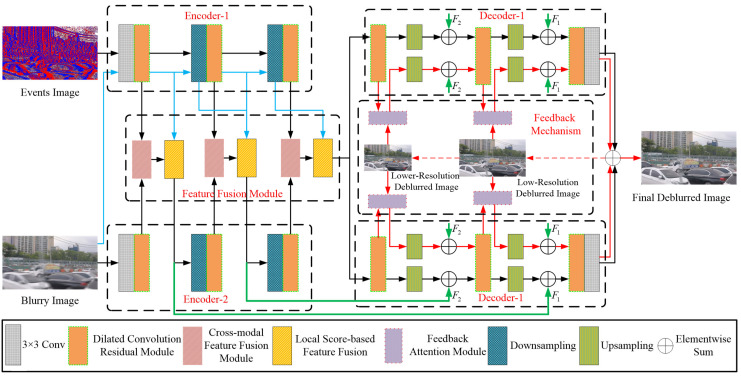
Complete network architecture for image deblurring.

To fully exploit the high temporal resolution of event cameras, this paper proposes an image deblurring model that fuses event and frame images. This model guides the restoration of dynamic blur regions in blurred images. The complete model consists of two parts, as illustrated in Figure 1. The first part comprises Encoder 1, Encoder 2, and a feature fusion module. Encoder 1 and Encoder 2 have the same architecture. Each encoder consists of a 3 × 3 convolution, a Dilated Convolutional Residual Module (DCRM), and a downsampling module. Encoder 1 is responsible for extracting event features. Encoder 2 extracts image features. Two downsampling operations in encoder are applied. They progressively reduce the spatial resolution to 1/2 and 1/4 of the original size. This captures richer structural information. The two encoders extract features, which are fused in the feature fusion module. A fusion mechanism is designed in this module. It consists of the Cross-Modal Feature Fusion Module (CFFM) and Local Score-based Feature Fusion (LSFF). This mechanism achieves efficient fusion and optimization of multi-modal, multi-scale features. Specifically, the CFFM module fuses the feature maps output by Encoder 1 and Encoder 2 at each scale. A cross-modal attention mechanism is incorporated. This achieves feature alignment and the integration of complementary information. It enhances the representational quality of the fused features.

Subsequently, the fused features output by the CFFM module are combined with the original outputs from the image feature encoder at the corresponding scales. They are then fed into the LSFF module. The LSFF module employs learnable spatial weight distributions. It suppresses noise and enhances details in the fused features. This improves deblurring performance at the feature level. The entire image feature fusion path consists of three CFFM modules and three LSFF modules. These modules operate on the encoder output features at three different scales. This hierarchical fusion and filtering mechanism enables the network to balance local detail preservation with global structural awareness. It enhances the consistency and effectiveness of multi-scale information fusion. As a result, more accurate intermediate features are provided for subsequent image reconstruction.

The second part of the model consists of two symmetrical but oppositely directed decoders. These decoders progressively reconstruct the sharp image. The two decoders have the same architecture. This ensures consistent deep feature extraction across modalities at the same hierarchical level. Both decoders take the feature fusion module’s output as input. However, a rotation-sharing mechanism is introduced during decoding. This mechanism achieves complementary directional deblurring modeling. Specifically, it shares convolutional kernel parameters between the two decoders. Decoder 1 maintains the original orientation and models horizontal blur. The convolutional kernels of Decoder 1 are then spatially rotated. These rotated kernels form the kernels for Decoder 2, which models vertical blur. This strategy enhances the decoders’ ability to distinguish and respond to blur in different directions. As a result, more efficient and direction-aware image deblurring is achieved.

Both decoders consist of DCRM modules and upsampling modules. The DCRM modules are responsible for deep feature decoding and fusion. They extract mid-to-high-level semantic information. The upsampling modules restore the feature maps to the original image resolution. This is done through two successive upsampling operations. The mapping from feature space to image space is thus completed. The two decoder models blur from different directions. Their final outputs are aligned by rotation and added pixel-wise. This approach further enhances the network’s capability to model and restore blur from multiple directions. Unlike conventional single-decoder deblurring networks that adopt undirected, uniform feature reconstruction, our dual-decoder design introduces a rotation-shared convolutional kernel mechanism to explicitly model the directional blur characteristics inherent in real motion blurs. Specifically, the two decoders share identical network architectures but adopt rotated kernel distributions for complementary feature learning: one decoder maintains the original convolutional kernels to capture and restore horizontal motion blur features, while the other decoder employs spatially rotated kernels derived from the shared parameter set to model vertical blur degradation specially. This dual-direction modeling strategy enables the network to separately perceive and reconstruct blurred features along horizontal and vertical dimensions, rather than treating all blur patterns indiscriminately. It compensates for the limitation of single-branch networks in handling complex, non-uniform, and multi-orientation motion blurs, significantly reducing structural distortion, edge blurring, and incomplete detail recovery in real blurred scenes. The final pixel-wise fusion of dual-direction decoded features comprehensively integrates multi-orientation restoration results, thereby substantially strengthening the network’s overall capability for complex image deblurring.

Furthermore, a feedback attention module is introduced within the encoder. This module guides dynamic feature selection during the feature extraction stage. It feeds back key features extracted from the previous encoding stage to the current stage. Weighted fusion is then performed through an attention mechanism. This strengthens inter-stage information flow and contextual consistency. Compared to traditional unidirectional feedforward feature extraction, the feedback mechanism offers clear advantages. It enhances the network’s perception of blurred region structures. It also improves the granularity of deep semantic representations. As a result, the overall image deblurring performance is effectively improved.

### 3.2. Proposed Cross-Modal Feature Fusion Module (CFFM)

In event-assisted image deblurring tasks, the information captured by event cameras during the equivalent exposure time is highly correlated with the frame images. However, significant differences exist between the two modalities. These differences lie in sensing principles, dynamic range, and spatiotemporal distribution. They lead to modal inconsistencies in the feature space. This issue is frequently discussed in multimodal learning [26]. Nevertheless, in event-guided image restoration tasks, existing methods typically fuse features from the two modalities directly [27]. They use simple convolutions or attention mechanisms. The semantic-level and statistical distribution discrepancies between modalities are neglected. This often results in information redundancy or guidance bias. These issues negatively affect the final reconstruction quality.

**Figure 2:**
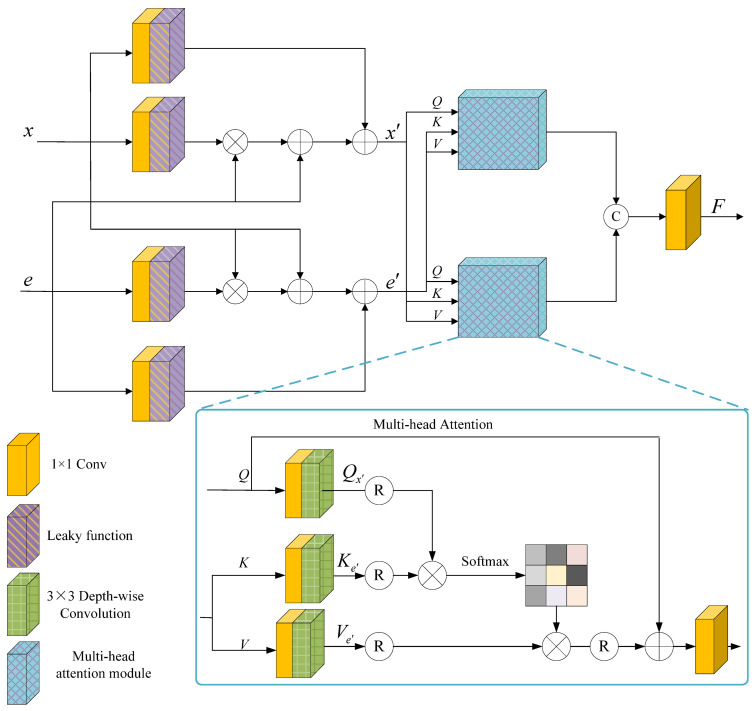
Cross-modal Feature Fusion Module (CFFM).

To address this problem, we proposed an effective Cross-modal Feature Fusion Module (CFFM). This module enhances feature fusion in a more coherent environment, as illustrated in Figure 2. The CFFM module consists of two main components. They are a feature alignment unit and a multi-head attention mechanism. During feature alignment, CFFM employs convolutional operations. These operations guide the features of one modality to participate in the transformation of the other modality’s features. This bridges the gap caused by inter-modal inconsistency. The frame image features and event image features are mutually adjusted. A combination of addition and multiplication operations is used. This compensates for representation shifts caused by modal discrepancies. The aligned features are then fed into the attention module. The attention mechanism plays a bidirectional role here. Frame features serve as the query vectors (Q) to extract key responses (K,V) from event features. Conversely, event features can also query frame features in the same manner. This enables cross-guidance between the two modalities. It also aligns salient regions.

Since the attention weights are shared, motion information can be fused within a more coherent and unified feature space. The CFFM module can be formulated as follows:

(1) $$x^{\prime }=Conv(x)+(Conv(x)\times e+e)$$

(2) e′=Conv(e)+(Conv(e)×x+x)

(3) $$F=Conv(Cat(Att(Q_{x^{\prime }},K_{e\prime },V_{e\prime }),Att(Q_{e\prime },K_{x^{\prime }},V_{x^{\prime }})))$$

where x denotes the image features, e denotes the event features, $x^{\prime }$ and e′ represents the aligned image and event features, respectively. Conv() denotes convolution operation. Q, K and V denote three inputs of the multi-head attention module. $Q_{x^{\prime }}$, $K_{x^{\prime }}$ and $V_{x^{\prime }}$ denote the query, key, and value matrices computed from the aligned image features $x^{\prime }$. $Q_{e\prime }$, $K_{e\prime }$ and $V_{e\prime }$ are the corresponding matrices derived from the aligned event features e′. These matrices are used to perform the multi-head attention operations. Att() denotes the multi-head attention operation, Con() represents the concatenation along the channel dimension, and F denotes the final output of the module.

### 3.3. Proposed Local Score-Based Feature Fusion Module (LSFF)

**Figure 3:**
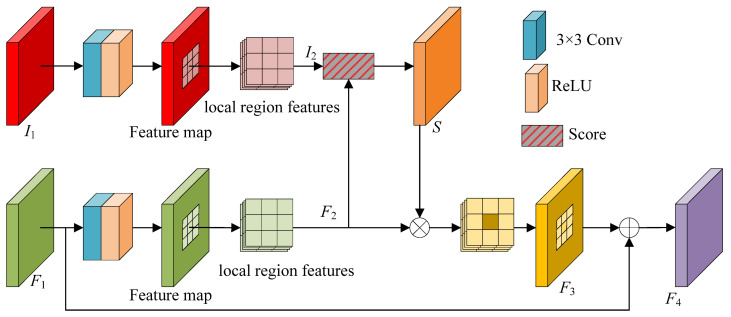
Local Score-based Feature Fusion (LSFF).

Event cameras inherently possess noise characteristics. Their response mechanism also differs from that of frame-based cameras. This is because they are fundamentally different types of imaging devices. Consequently, the pixels triggered by events do not necessarily correspond to blurred regions in the image. To address this issue, we design a Local Score-based Feature Fusion (LSFF) module and propose a Dilated Convolution Residual Module. This module identifies regions where events occur and blur is likely to exist. At the same time, it suppresses irrelevant event areas. The LSFF module is illustrated in Figure 3. Specifically, we feed the image features $I_{1}$ and the spatially aligned fused features $F_{1}$ into a 3 × 3 convolutional layer with ReLU activation to obtain a feature map of size $R^{\hat{C}\times \hat{W}\times \hat{H}}$. Then, for each pixel location, we sample local neighborhood patches from both the image features and event features using a predefined window, resulting in $I_{2},F_{2}\in R^{M\times \hat{C}\times \hat{W}\times \hat{H}}$, which represent the local region features around each spatial location from the image and event modalities, respectively (where M denotes the number of neighboring pixels). Subsequently, we compute the similarity between $I_{2}$ and $F_{2}$ to generate a matching score:

(4) $$S=\langle I_{2},F_{2}\rangle \in R^{M\times \hat{H}\times \hat{W}},$$

where $\langle \cdot ,\cdot \rangle$ denotes the inner product operation. The resulting score map encodes the similarity between the bimodal representations, effectively suppressing redundant event responses in the deblurring task. Using the computed similarity scores and the aligned features of each neighborhood pixel, we perform weighted aggregation of the features as follows:

(5) $$F_{3}(\rho )=\sum_{i=1}^{n}F_{2}(i,\rho )\times sig(S(i,\rho )),$$

where $F_{2}(i,\rho )$ denotes the spatially aligned event feature sampled at the ith neighborhood around position ρ, and S(i,ρ) represents the similarity score between the image and event features at that *i*th neighborhood location. sig() denotes the Sigmoid function, which maps the similarity scores to the range (0, 1). n indicates the number of neighboring pixels involved in the weighted computation. $F_{3}(\rho )$ denotes the final aggregated feature at position ρ, obtained through the weighted fusion.

Finally, the output of the LSFF module is obtained by adding the aggregated feature $F_{3}(\rho )$ and the spatially aligned event feature $F_{1}$ is expressed as:

(6) $$F_{4}=F_{3}+F_{1},$$

By comprehensively considering neighborhood information, the LSFF module obtains more reliable fused features. Even in the presence of repetitive structural patterns in the image, this module effectively alleviates uncertainty and ambiguity between modalities.

### 3.4. Proposed Dilated Convolution Residual Module (DCRM)

**Figure 4:**
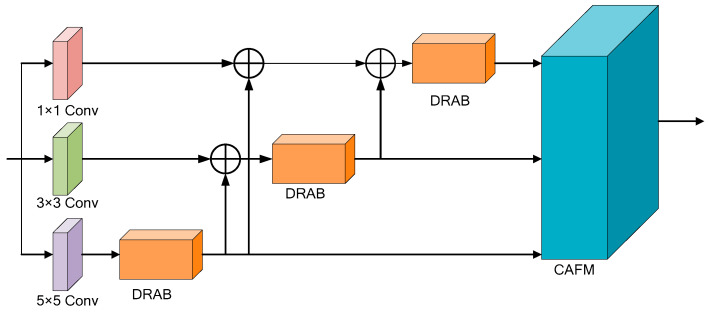
Dilated convolution residual module (DCRM).

**Figure 5:**
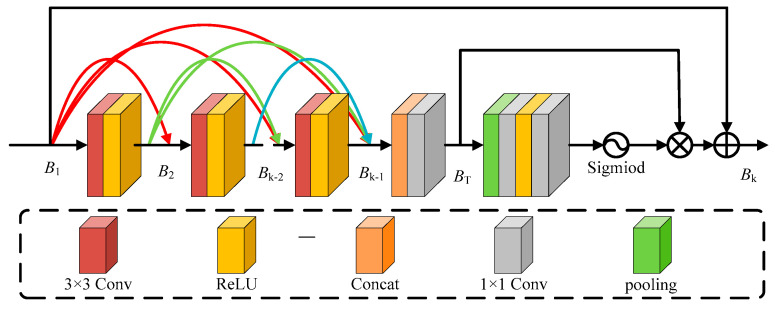
Dense Residual Attention Block (DRAB).

**Figure 6:**
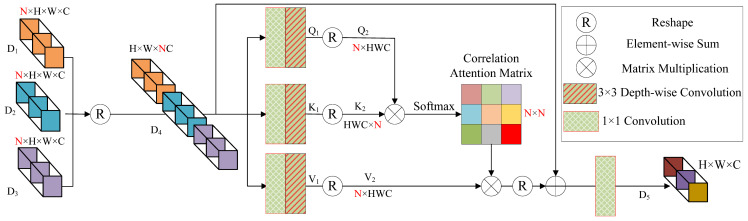
Cross-Layer Attention Fusion Module (CAFM).

To extract richer features from blurred images, we propose a Dilated Convolution Residual Module (DCRM). This module is illustrated in Figure 4. Specifically, we employ parallel dilated convolutions. The dilation rates are 1, 3, and 5. These convolutions first extract multi-scale features from the image. Dilated convolutions have a clear advantage. They expand the receptive field without introducing many parameters. Subsequently, we introduce our designed Dense Residual Attention Block (DRAB) modules on each branch. The DRAB modules are shown in Figure 5. They further extract image features. Progressive residual connections are incorporated to enhance model performance. Finally, we use the Cross-Layer Attention Fusion Module (CAFM) to further fuse the features. The CAFM module is shown in Figure 6.

#### 3.4.1. Proposed Dense Residual Attention Block (DRAB)

To address the common issues of insufficient feature extraction and detail loss in low-resolution images, we design a Dense Residual Attention Block (DRAB). Its architecture is shown in Figure 5. This module consists of two key components. They are a Dense Residual Block and a Channel Attention mechanism. First, we construct a dense residual block composed of three basic blocks, each containing convolutional layers followed by ReLU activation, to extract feature maps. Then, through feature concatenation and a 1 × 1 convolution, the features extracted by each convolutional layer are merged, and the channel dimension is reduced to produce the feature map. Finally, a channel attention mechanism is employed to learn the importance of different feature channels, while residual connections are used to enhance the model’s performance.

Let the input and output of the dense residual block be $B_{1}$ and $B_{k}$, respectively. The computation process of the dense residual block is described as:

(7) Bk−1=Re(Conv3(cat([B1,…,Bk−2]))),

where $Conv_{3}()$ denotes convolution operation with kernel size 3. Cat() denotes the feature concatenation operation. Re() denotes the ReLU activation function, and $B_{k-1}$ is the feature map output by the convolution. The features extracted from each layer are then fused as:

(8) $$B_{T}=Conv_{1}(cat([B_{1},\ldots ,B_{k-1}])),$$

where $Conv_{1}()$ denotes convolution operation with kernel size 3, and Cat() denotes the feature concatenation operation. The fused features are then fed into the channel attention module. First, global average pooling is applied to perform global compression, resulting in a vector z, as described in (9):

(9) $$z=H_{GP}(B_{T})=\frac{1}{H\times W}\sum_{i=1}^{H}\sum_{j=1}^{W}B_{T}\left(i,j\right),$$

where $H_{GP}$ denotes the adaptive average pooling operation, $B_{T}(i,j)$ represents the channel vector $B_{T}$ at the spatial position (i,j). Then, a dimensionality reduction fully connected layer, followed by a ReLU activation function, is applied. After upsampling the output to the original channel dimension, Sigmoid is utilized to generate channel-wise attention weights, as given in (10):

(10) W=Sig(conv1(Re(conv1(z)))),

where sig() denotes the Sigmoid function, Re() denotes the ReLU activation function. Finally, the output feature $B_{k}$ of the DRAB module, as shown in (11):

(11) $$B_{k}=B_{k-1}+W\times B_{T}$$

#### 3.4.2. Proposed Cross-Layer Attention Fusion Module (CAFM)

In recent years, numerous deep learning-based image restoration approaches integrate multi-scale and multi-semantic features via feature concatenation or skip connections. Nevertheless, such fusion schemes fail to adequately model dependencies across hierarchical features, thereby restricting the model’s feature representation capabilities. To tackle this limitation, we design a novel Cross-Layer Attention Fusion Module (CAFM) that adaptively aggregates multi-level features with learnable attention weights. The core principle of CAFM is that activation outputs from distinct layers correspond to feature embeddings for specific semantic content; the self-attention mechanism precisely captures inter-layer dependencies and further enhances the comprehensive representation ability of fused features.

As shown in Figure 6, given the concatenated features from N consecutive layers, denoted as $D\in R^{N\times H\times W\times C}$. We first concatenate the three input features $D_{1}$, $D_{2}$ and $D_{3}$, and reshape them into a H×W×(N×C) dimensional feature tensor $D_{3}$. Next, we apply a 1 × 1 convolution to aggregate information across the channel dimension at each pixel location to extract pixel-level cross-channel contextual features. Then, a 3 × 3 depthwise separable convolution is used to generate the Query (Q_1_), Key (K_1_), and Value (V1) matrices. Subsequently, the Query and Key are reshaped into two-dimensional matrices of size $Q_{1}\in R^{N\times (H\times W\times C)}$ and $K_{1}\in R^{(H\times W\times C)\times N}$, respectively, and the inter-layer correlation attention matrix A of size N×N is computed. Finally, the reshaped Value matrix $V_{1}\in R^{(H\times W\times C)\times N}$ is multiplied by the attention matrix A (with scaling factor α) and then added to the input feature $D_{4}$. The processing of CAFM is defined as follows:

(12) $$D_{4}=cat(D_{1},D_{2},D_{3}),$$

(13) $$D_{5}=Conv_{1}((Cam(Sof(Q_{2}\times K_{2})))\times V_{2}+D_{4})$$

where $Conv_{1}()$ denotes the convolution operation with kernel size 3, and Cat() denotes the feature concatenation operation. Cam() denotes the correlation attention matrix. Sof() denotes the softmax function, which normalizes the inter-layer similarity matrix to generate attention weights.

### 3.5. Proposed Feedback Attention Module (FAM)

In image deblurring tasks, low-level features typically contain rich texture and edge information, while high-level features capture more abstract semantic representations. To more effectively leverage high-level semantic feedback from deeper decoders to guide the restoration of texture details in shallow decoders, we propose a Feedback Attention Module (FAM). This module integrates feedback from higher layers with current-layer representations to guide the network’s focus toward more informative image regions, thereby improving image reconstruction quality.

**Figure 7:**
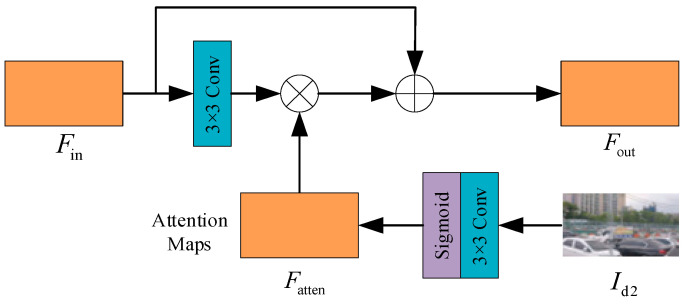
Feedback Attention Module (FAM).

The FAM module is integrated into each layer of the decoder to adaptively select key features from the feedback path and enhance the representation of the current features. Its structure is illustrated in Figure 7. Taking the bottom layer of the decoder as an example, let the input feature of the current layer be denoted as $F_{in}$, and the feedback information from the upper layer be denoted as $I_{d2}$. First, a 3 × 3 convolution is applied to the feedback information $I_{d2}$, followed by a Sigmoid activation function to generate an attention map $F_{\mathrm{atten}}$, which represents the importance weights of the feedback features at different spatial locations. This attention map adaptively reflects the significance of different spatial positions, thereby highlighting the informative regions beneficial to the current input feature and effectively guiding the enhancement and reconstruction of the current layer’s features. Subsequently, the current input feature is passed through a 3 × 3 convolutional layer and element-wise multiplied with the attention map to obtain the attention-modulated feature response. This response is then added to the original input to produce the final output, completing the feedback enhancement process.

This process not only integrates multi-level semantic information and local details but also avoids redundant features caused by simple concatenation. Especially in deblurring tasks, features in blurred regions are often unstable or severely degraded, while high-level semantic feedback can provide contextual compensation. The FAM module “feeds back” high-level information to lower layers in the form of “attention”, enhancing their ability to model fine details. This is particularly effective in restoring structural blur and unclear edge regions. The detailed formulation is as follows:

(14) $$F_{\mathrm{atten}}=Sig(Conv_{3}\left(I_{d2}\right)),$$

(15) $$F_{out}=F_{atten}\times Conv_{3\times 3}\left(F_{in}\right)+F_{in},$$

where Sig() denotes the Sigmoid activation function, and $Conv_{3}$ represents a 3 × 3 convolution operation.

### 3.6. Proposed Loss Function

To effectively guide the network in restoring clear textures and structural information from severely blurred images, we design a composite loss function that jointly considers pixel accuracy, structural similarity, and frequency-domain consistency. The overall loss function is defined as follows:

(16) $$L_{total}=\lambda_{1}L_{MAE}+\lambda_{2}L_{SSIM}+\lambda_{3}L_{MSFR},$$

where λ denotes the importance weights of each loss function, set as $\lambda_{1}=0.7$, $\lambda_{2}=0.2$, $\lambda_{3}=0.1$. The Mean Absolute Error (MAE) loss is used to measure the pixel-level difference between the predicted image $P_{i}$ and the ground truth image $T_{i}$, and is defined as:

(17) $$L_{MAE}=\frac{1}{N}\sum_{i=1}^{N}{\left\|P_{i}-T_{i}\right\|}_{1},$$

where N represents the total number of pixels in the image. The loss assigns equal weight to each pixel’s error, providing stable gradients across the entire image and being especially effective for restoring blurred regions.

To improve the structural and perceptual quality of images, this paper introduces the Structural Similarity (SSIM) loss, which measures the similarity between the predicted and ground-truth images across three components: luminance, contrast, and structure. It is defined as follows:

(18) $$L_{SSIM}=1-SSIM\left(P,T\right),$$

where a higher SSIM value indicates greater structural similarity, with a maximum value of 1. By minimizing 1 − SSIM, the network is encouraged to generate clearer structural details that better align with human visual perception.

To further enhance the image’s ability to preserve details, especially high-frequency texture information, this paper introduces the Multi-Scale Frequency Reconstruction (MSFR) loss [28]. Unlike pixel-wise losses that only constrain spatial pixel errors, this loss computes spectral discrepancies across multi-scale images via Fourier transforms. This loss calculates the differences in the image spectra at different scales through Fourier transforms and is defined as follows:

(19) $$L_{MSFR}=\sum_{s=1}^{S}\frac{1}{N_{s}}{\left\|F\left(P(s)\right)-F\left(T(s)\right)\right\|}_{1},$$

where $F\left(\cdot \right)$ denotes the discrete Fourier transform, P(s) and T(s) represent the predicted image and the ground truth image at the s-th scale, respectively. $N_{s}$ is the total pixel number of the s-th scaled image. Conventional L1/MSE loss uniformly constrains all pixels in the spatial domain, treating smooth low-frequency backgrounds and edge/texture high-frequency components equally. This uniform supervision easily suppresses tiny textures and edges, leading to over-smoothed, blurry results.

In contrast, the MSFR loss transforms images into the frequency domain at every scale and minimizes the spectral gap between restored results and ground truth. High-frequency Fourier coefficients capture critical fine textures, contours, and tiny structural details, while low-frequency components correspond only to flat background areas. By explicitly penalizing mismatches in full-spectrum signals across multi-scale layers, this loss imposes stronger supervision on high-frequency information. It forces the network to accurately fit the high-frequency spectrum of sharp ground truth, retaining delicate textures and sharp edges, and alleviates the over-smoothing artifact prevalent in traditional deblurring methods.

## 4. Simulation and Discussion

For evaluation, we trained the model on the GoPro dataset [15], which contains 3214 pairs of blurred and sharp images. We selected 2103 pairs and augmented the data with random horizontal and vertical flips, resulting in 4206 image pairs used as the training set. Additionally, to further test the model’s deblurring performance, we evaluated it on the GoPro dataset, REBlur dataset [29] and the RwEvent dataset [30]. We used the Ubuntu 18.04 system in our experiment. The GPU was an NVIDIA GeForce GTX 2080Ti. The deep learning framework was PyTorch (version PyTorch 1.6.0). During training, the number of epochs was set to 1000, the batch size was 4, and the model was optimized using the AdamW optimizer with cosine annealing. The initial learning rate was set to 2 × 10^−4^ and gradually decreased to 1 × 10^−7^ using a cosine annealing schedule.

### 4.1. Comparison on the GoPro Dataset

**Figure 8:**
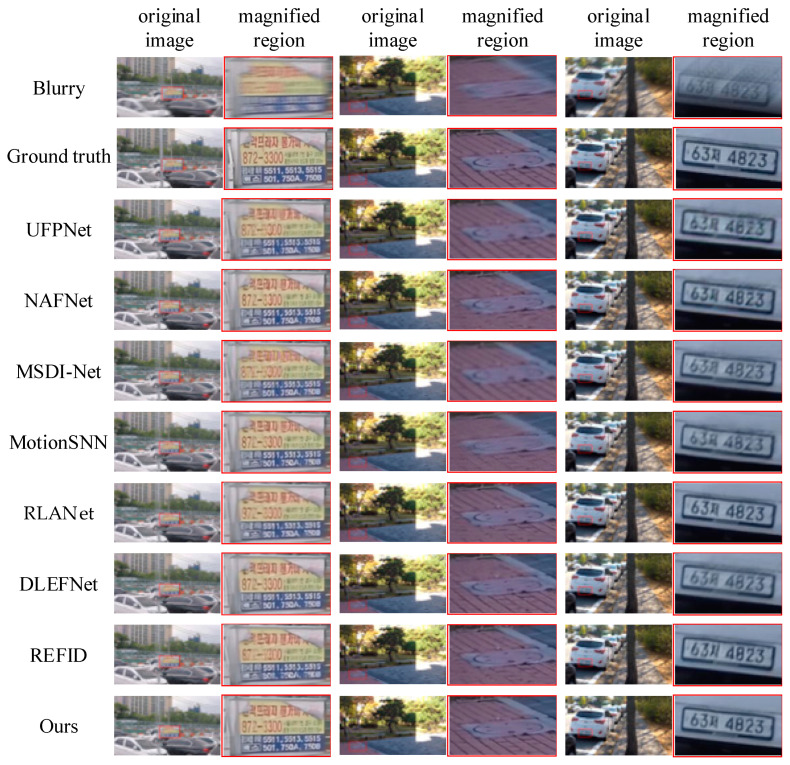
Qualitative comparison in the GoPro dataset.

We qualitatively evaluate our method on the GoPro dataset. We compare its performance with existing algorithms, namely UFPNet [12], MSDI-Net [13], MotionSNN [18], RLANet [20], NAFNet [31], REFID [32], DLEFNet [33]. Figure 8 displays all comparison results. Three image groups are selected for visual comparison. Local regions are magnified to highlight visual differences. From Figure 8, obvious defects appear in the first image group. UFPNet, MSDI-Net, and RLANet produce fuzzy, illegible text in the first line. The MotionSNN result only reserves the upper triangular part of letter “A” in the bottom text line. The original shape of “A” is largely destroyed. The number “7508” at the bottom-right corner is hard to distinguish in the outputs of RLANet and REFID. Our method restores these fine details with sharpness and completeness. Overall, our proposed method outperforms all compared competitors. In contrast, our method recovers such fine details distinctly. Among all competing schemes, the proposed method delivers optimal deblurring performance and sharper image textures.

For the second test group, UFPNet, MSDI-Net and MotionSNN retain prominent blur artifacts. NAFNet and DLEFNet fail to restore distinct white speckles on the manhole cover. Our method reconstructs these speckles with high fidelity. In the third group, severe blur obscures license plate characters in outputs of UFPNet, MSDI-Net, MotionSNN, NAFNet and DLEFNet. RLANet introduces structural distortion compared with ground truth. REFID produces noticeable shape deformation for the symbol behind digit “3”. These visual comparisons further verify the superior deblurring capability of our proposed method.

We adopt quantitative metrics to validate the efficacy of our method objectively. We test all paired samples from the GoPro test set and calculate average peak signal-to-noise ratio (PSNR), average structural similarity index measure (SSIM), parameter counts (Para, M), giga floating-point operations (GFLOPs) and inference frames per second (FPS) for all comparative approaches. All quantitative results are summarized in Table 1. Table 1 reports the full comparison results of UFPNet, NAFNet, MSDI-Net, MotionSNN, RLANet, DLEFNet, REFID and our method on the GoPro dataset. In terms of restoration accuracy, our method achieves a maximum PSNR of 36.13 dB and a peak SSIM of 0.975. REFID ranks second with a PSNR of 35.90 and SSIM of 0.974, while DLEFNet takes the third place (35.61 PSNR, 0.974). Among all competitors, MSDI-Net yields the lowest PSNR (33.28) and SSIM (0.964). From the model-scale perspective, our model contains only 17.6 M parameters, which is far lighter than heavy architectures such as UFPNet (80.3 M), NAFNet (67.8 M), and MSDI-Net (135.4 M). In terms of computational complexity, our GFLOPs are 1089.01, much lower than those of UFPNet and MSDI-Net. In terms of real-time inference speed, DLEFNet achieves the highest FPS at 12.79, followed by REFID (12.18) and our method (10.06). Despite not being the fastest inference model, our approach achieves the best image restoration performance with a moderate parameter size and computational cost. Table 1 comprehensively demonstrates that our method achieves state-of-the-art restoration accuracy while maintaining lightweight model overhead.

### 4.2. Comparison on REBlur and RwEvent Datasets

**Figure 9:**
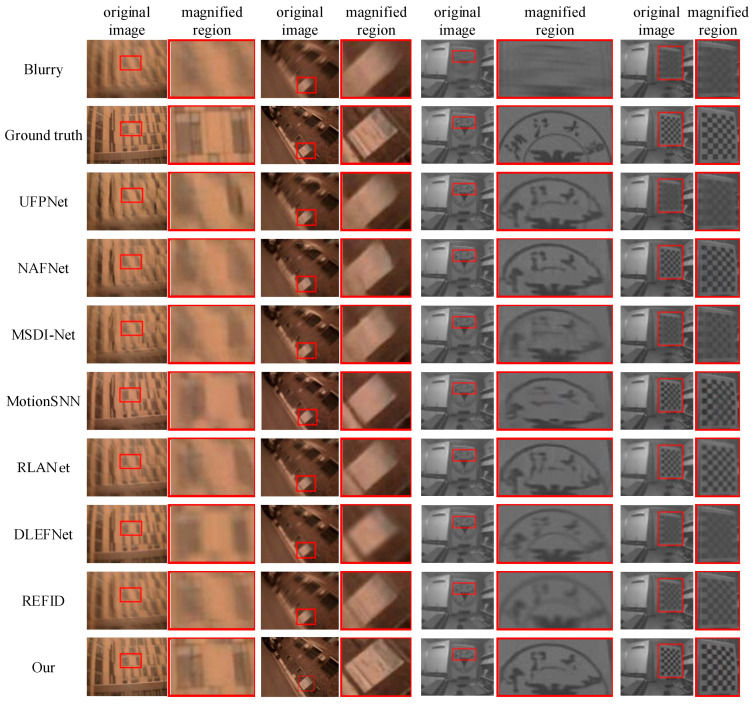
Qualitative comparison on REBlur and RwEvent datasets.

Qualitative evaluations of the proposed algorithm are carried out on the ReBlur and RwEvent datasets separately. We compare its performance against other competing algorithms. Four image groups are selected for qualitative comparison. Figure 9 illustrates all comparative visual results. Local regions are magnified to facilitate intuitive visual contrast. For the first scene of building windows: UFPNet, NAFNet, MSDI-Net, MotionSNN, DLEFNet, RLANet, and REFID produce severely blurred outputs. Texture loss and blurred edges occur in window areas, resulting in hazy results. Window structures and fine details are barely recognizable. By contrast, our method accurately reconstructs complete window contours and textures. Frame and pane details are fully preserved with greatly improved visual distinguishability. For the second billboard scene, all baselines, except for REFID and our method, restore the billboard regions as nearly blank white areas. Text and pattern information on billboards are completely lost. REFID can only render faint, blurry traces with low recognizability. Our approach reconstructs clearer textures and marks on billboards. It achieves higher integrity and sharpness than REFID.

For the third text sign scene, MSDI-Net, DLEFNet, and REFID suffer from obvious blurring artifacts. Text edges become fuzzy, and strokes stick together. MotionSNN and RLANet cause severe text shape distortion and stroke deformation. Text content cannot be identified correctly. Our method recovers intact stroke structures and shapes for legible text. For the fourth structured-grid scene, UFPNet, NAFNet, MSDI-Net, MotionSNN, DLEFNet, RLANet, and REFID exhibit prominent blur and texture distortion. Grid boundaries blur and overlap. Our method precisely restores sharp grid boundaries and regular textures with optimal detail fidelity. In summary of qualitative evaluation on the four test groups: The proposed deblurring algorithm outperforms all comparative methods in detail reconstruction, texture preservation and visual clarity. It achieves state-of-the-art deblurring performance and sharper restored images.

We conduct quantitative tests to verify the effectiveness of our method objectively. All paired samples of REBlur and RwEvent are adopted for evaluation. Average PSNR and SSIM values are calculated for each method. Table 2 summarizes these quantitative results. We further explore the generalization performance on diverse blurred scenes. Table 2 lists numerical metrics on the two real-world datasets. Our model achieves the maximum PSNR and SSIM on both datasets. It ranks first in PSNR and SSIM for both REBlur and RwEvent. This outcome verifies its powerful generalization against real motion blur. The model works well under complicated non-uniform blur distributions. It accurately recovers fine textures and structural information.

### 4.3. Ablation Study

Ablation experiments are implemented to verify the validity of four core components. These components include the cross-modal feature fusion module, the local score-based feature fusion module, the dilated multi-scale residual module, and the feedback attention module.

Four ablation variants are defined as follows:

w/o CFFM: Remove the cross-modal Feature Fusion Module; only simple ordinary cross-attention is adopted.

w/o LSFF: Remove the Local Score-based Feature Fusion module.

w/o DCRM: Remove the Dilated Convolution Residual Module; only common convolution is adopted.

w/o DRAB: Remove the DRAB from DCRM.

w/o FAM: Remove the Feedback Attention Module.

w/o SRK: Remove the sharing rotated-kernel.

w/o MSFR: Remove the Multi-Scale Frequency Reconstruction (MSFR) loss.

All paired test images from the GoPro dataset are used for evaluation. We compute PSNR and SSIM for each ablation variant. Quantitative results are listed in Table 3. The CFFM bridges the distribution gap between image and event modalities and realizes reliable cross-modal feature fusion. After removing CFFM, the PSNR declines from 36.13 to 34.61, and the SSIM drops from 0.975 to 0.958, resulting in the largest performance degradation across all variants and validating that cross-modal feature alignment is the most vital component for high-fidelity blur removal.

The DCRM captures multi-scale motion-blur patterns via dilated convolutional branches to enlarge effective receptive fields. Without DCRM, the PSNR falls to 34.68 and SSIM reduces to 0.961, revealing that multi-scale motion modeling greatly enhances reconstruction accuracy and edge preservation. The LSFF suppresses background noise and highlights motion-corrupted regions via local feature matching. Eliminating LSFF reduces PSNR to 35.02 and SSIM to 0.965, demonstrating that local spatial filtering eliminates noisy event signals and stabilizes structural restoration.

The MSFR aggregates multi-layer motion features to enrich fine texture representations. Removing MSFR yields a PSNR of 35.16 and an SSIM of 0.966, demonstrating that multi-scale feature recalibration supplements missing texture information in blurry regions. The DRAB adaptively assigns weights to motion features according to local blur intensity. When DRAB is excluded, PSNR reduces to 35.73 and SSIM to 0.968, indicating that dynamic motion weighting can better distinguish blurred areas from static backgrounds. The FAM delivers high-level semantic cues to shallow layers for texture compensation. Discarding FAM results in PSNR of 35.89 and SSIM of 0.970, which verifies that semantic feedback recovers subtle structural details lost during downsampling. The SRK module conducts weight-sharing rotation convolution to handle oblique motion blur. Without SRK, the PSNR drops only slightly to 35.94, and the SSIM decreases to 0.973. This minor metric gap indicates that SRK delivers steady yet moderate performance gains in slanted-blur scenarios.

In addition to quantitative restoration metrics, we further analyze the performance trade-offs of each ablation variant. Removing any single module reduces model complexity marginally, yet the clear PSNR and SSIM declines demonstrate that lightweight gains come at the cost of degraded deblurring quality. CFFM delivers the most significant performance improvement with manageable computational overhead, while DCRM, LSFF, MSFR, DRAB, FAM, and SRK continuously refine pixel accuracy and structural integrity with limited additional computational burden. Our full model achieves the optimal balance between restoration quality and computational overhead.

Moreover, we supplement the analysis of overfitting risk as suggested by the reviewer. During training, we adopt random horizontal and vertical flip augmentation, Adam optimizer with weight decay, and cosine annealing learning rate scheduling to regularize the network. We continuously track training and validation PSNR and SSIM throughout the whole training process. No sustained widening of the gap between training and validation metrics is observed, indicating that neither the full model nor any ablation variant suffers severe overfitting under our training setup.

In conclusion, every proposed module provides consistent positive gains in both PSNR and SSIM. Integrating all seven modules jointly yields the optimal pixel reconstruction and structural similarity performance on the GoPro dataset.

### 4.4. Limitations and Failure Cases

Although the proposed network achieves competitive deblurring performance on the GoPro, REBlur, and RwEvent datasets, there are noticeable limitations and typical failure cases that require further optimization. The dual-decoder structure can model horizontal and vertical blur separately, yet for scenes with arbitrary diagonal ultra-fast motion, the fixed kernel rotation strategy cannot fully fit irregular slant blur trajectories, leading to slight edge smearing on thin linear objects such as power lines and slender traffic signs. The LSFF module suppresses irrelevant background event signals via local similarity scoring, but when the scene contains extremely strong ambient noise that generates massive false event triggers, the bimodal matching score is disrupted, leading to incomplete removal of motion artifacts in fine textures. The model is mainly trained on daytime blurred samples with sufficient illumination. In night environments, the low signal-to-noise ratio of frame images reduces the reliability of reference features, weakening the cross-modal alignment effect of CFFM and lowering overall restoration quality.

Despite only 17.6 M parameters, the multi-scale hierarchical fusion pipeline involves repeated feature aggregation operations. When processing images larger than 1920 × 1080 on an RTX 2080 Ti, the FPS drops significantly, limiting real-time deployment for ultra-high-definition video.

When small objects move rapidly along diagonal directions, horizontal and vertical decoding branches only handle two orthogonal blur directions. The residual slant blur cannot be fully decomposed, so the restored object boundaries remain fuzzy and lack sharpness. Low-light frames lose abundant texture details, which breaks the bimodal feature matching basis of CFFM. Event streams become the dominant input, yet event intensity information is incomplete, resulting in over-smoothed restored images with missing subtle textures.

## 5. Conclusions

A novel dual-encoder dual-decoder network architecture is proposed in this work to address the image deblurring problem. The network integrates three functional modules: the cross-modal feature fusion module, the local score-based event fusion module, and the dilated convolutional residual module. On this basis, we construct a complete image deblurring framework. To further elevate deblurring accuracy, we adopt a dual-decoder structure. Meanwhile, a feedback mechanism is introduced into the encoder–decoder pipeline, enabling adaptive selection of discriminative features along feedback paths and yielding remarkable gains in image reconstruction. Our model is trained on the training subset of the GoPro dataset and evaluated on GoPro, REBlur, and RwEvent datasets. PSNR and SSIM are used to assess model performance quantitatively. Experimental results show that our method achieves the highest PSNR and SSIM values on three datasets, demonstrating its competitive advantages over peer algorithms. In addition, ablation analyses verify the effectiveness of each module. As demonstrated by comprehensive experiments, the proposed method outperforms existing solutions in both visual quality and quantitative metrics for image deblurring. For future work, we will further optimize the network architecture by combining lightweight design strategies and efficient attention mechanisms to reduce computational overhead and accelerate high-resolution inference. We also plan to enhance the model’s generalization and robustness for complex real-world blurred scenes. Moreover, we will conduct in-depth research on model quantization and deployment optimization and extend the proposed deblurring algorithm to embedded devices and real-time video deblurring tasks to broaden its practical application scenarios.

## Figures and Tables

**Table 1:** Quantitative comparison on the GoPro dataset.

	UFPNet	NAFNet	MSDI-Net	MotionSNN	RLANet	DLEFNet	REFID	Our
PSNR	34.06	33.71	33.28	35.18	34.25	35.61	35.90	36.13
SSIM	0.968	0.967	0.964	0.971	0.953	0.974	0.974	0.975
Para (M)	80.3	67.8	135.4	27.8	17.8	13.9	14.2	17.6
GFLOPs	4968.56	4195.13	8377.88	1843.88	1119.94	860.06	983.81	1089.01
FPS	2.21	2.62	1.31	5.97	9.82	12.79	12.18	10.06

**Table 2:** Quantitative comparison on REBlur and RwEvent datasets.

Method	REBlur		RwEvent	
	PSNR	SSIM	PSNR	SSIM
UFPNet	36.11	0.968	32.82	0.906
NAFNet	36.15	0.969	33.83	0.914
MSDI-Net	36.14	0.968	31.42	0.859
MotionSNN	36.32	0.968	33.87	0.907
RLANet	37.12	0.970	34.64	0.925
DLEFNet	38.40	0.976	35.10	0.933
REFID	38.34	0.975	34.88	0.923
Our	38.54	0.977	35.37	0.940

**Table 3:** Quantitative evaluation of ablation studies on GoPro dataset.

	w/o CFFM	w/o LSFF	w/o DCRM	w/o DRAB	w/o FAM	w/o SRK	w/o MSFR	Ours
PSNR	34.61	35.02	34.68	35.73	35.89	35.94	35.16	36.13
SSIM	0.958	0.965	0.961	0.968	0.970	0.973	0.966	0.975

## Data Availability

The data presented in this study are available upon reasonable request from the corresponding author.

## References

[1] Chen Y., Liu Y., Wang X., Zhang Q., Zhang X., Cao K., Jung H. (2026). Frequency-Aware Bidirectional Interactive Mamba Network for Image Super-Resolution. Electronics.

[2] Shehzadi T., Ifza I., Liwicki M., Stricker D., Afzal M.Z. (2026). Semi-Supervised Object Detection: A Survey on Progress from CNN to Transformer. Sensors.

[3] Guo Y., Ma L., Zhang Y. (2026). Image Deblurring via Frequency-Domain Feature Enhanced Convolutional Neural Networks. Sensors.

[4] Shi C., Jiang X., Zhang X., Zhu C., Hu X., Zhang G., Li Y., Zhang C. (2025). Real-Time Multi-Scale Barcode Image Deblurring Based on Edge Feature Guidance. Electronics.

[5] Wu L., Li M., Wang J., Li J., Zhou F., Zhu J. (2026). A Hough Transform-based calibration approach for event camera. Measurement.

[6] Ding S., Zhang H., Zhang Y., Huang X., Song W. (2025). Hyper real-time flame detection: Dynamic insights from event cameras and FlaDE dataset. Expert. Syst. Appl..

[7] Cao R., Galor D., Kohli A., Yates J.L., Waller L. (2025). Noise2Image: Noise-enabled static scene recovery for event camera. Optica.

[8] Ma Y., Kong D. (2024). Super-resolution reconstruction algorithm for dim and blurred traffic sign images in complex environments. AIMS Math..

[9] Wei Y.J., Di X. (2024). Method for simultaneous reconstruction of a depth and clear image with a single blurred image in microscopy. Appl. Opt..

[10] Ho Q.-T., Duong M.-T., Lee S., Hong M.-C. (2025). Adaptive Image Deblurring Convolutional Neural Network with Meta-Tuning. Sensors.

[11] Sun J., Cao W., Xu Z., Ponce J. (2015). Learning a convolutional neural network for non-uniform motion blur removal. Proceedings of the IEEE Conference on Computer Vision and Pattern Recognition, Boston, MA, USA, 7–12 June 2015.

[12] Fang Z., Wu F., Dong W., Li X., Wu J., Shi G. (2023). Self-supervised non-uniform kernel estimation with flow-based motion prior for blind image deblurring. Proceedings of the 2023 IEEE/CVF Conference on Computer Vision and Pattern Recognition (CVPR), Vancouver, BC, Canada, 17–24 June 2023.

[13] Li D., Zhang Y., Cheung K.C., Wang X., Qin X., Li H. (2022). Learning degradation representations for image deblurring. European Conference on Computer Vision.

[14] Kupyn O., Budzan V., Mykhailych M., Mishkin D., Matas J. (2018). DeblurGAN: Blind Motion Deblurring Using Conditional Adversarial Networks. Proceedings of the 2018 IEEE/CVF Conference on Computer Vision and Pattern Recognition, Salt Lake City, UT, USA, 18–23 June 2018.

[15] Nah S., Kim T.H., Lee K.M. (2017). Deep Multi-scale Convolutional Neural Network for Dynamic Scene Deblurring. Proceedings of the 2017 IEEE Conference on Computer Vision and Pattern Recognition (CVPR), Honolulu, HI, USA, 21–26 July 2017.

[16] Tao X., Gao H., Shen X. (2018). Scale-Recurrent Network for Deep Image Deblurring. Proceedings of the 2018 IEEE/CVF Conference on Computer Vision and Pattern Recognition, Salt Lake City, UT, USA, 18–23 June 2018.

[17] Mueggler E., Gallego G., Rebecq H., Scaramuzza D. (2018). Continuous-Time Visual-Inertial Odometry for Event Cameras. IEEE Trans. Robot..

[18] Liu Z., Wu J., Shi G., Yang W., Dong W., Zhao Q. (2024). Motion-oriented hybrid spiking neural networks for event-based motion deblurring. IEEE Trans. Circuits Syst. Video Technol..

[19] Jiang Z., Zhang Y., Zou D., Ren J., Lv J., Liu Y. (2020). Learning Event-Based Motion Deblurring. Proceedings of the 2020 IEEE/CVF Conference on Computer Vision and Pattern Recognition (CVPR), Seattle, WA, USA, 13–19 June 2020.

[20] Chen H., Teng M., Shi B., Wang Y., Huang T. (2022). A residual learning approach to deblur and generate high frame rate video with an event camera. IEEE Trans. Multimed..

[21] Wang B., He J., Yu L., Xia G.-S., Yang W. (2020). Event enhanced high-quality image recovery. Proceedings of the Computer Vision–ECCV 2020: 16th European Conference, Glasgow, UK, 23–28 August 2020.

[22] Zhang X., Yu L. (2022). Unifying Motion Deblurring and Frame Interpolation with Events. Proceedings of the 2022 IEEE/CVF Conference on Computer Vision and Pattern Recognition (CVPR), New Orleans, LA, USA, 18–24 June 2022.

[23] Geng M., Wang L., Zhu L., Zhang W., Xiong R., Tian Y. (2025). Event-Enhanced Snapshot Mosaic Hyperspectral Frame Deblurring. IEEE Trans. Pattern Anal. Mach. Intell..

[24] Weng W., Zhang Y., Xu W., Xiao Z., Xiong Z. (2025). BVSR-EvD: Blurry Video Space-Time Super-Resolution with Events via Diffusion Models. IEEE Trans. Image Process..

[25] Fu Y., Zhou C., Chen W., Wu T., Li Q., Wu X. (2026). EDVD: Cross-Modal Spatio-Temporal Fusion with Event and Diffusion for Video Deblurring. IEEE Trans. Image Process..

[26] Li Y., Yu A.W., Meng T., Caine B., Ngiam J., Peng D., Shen J., Lu Y., Zhou D., Le Q.V. (2022). DeepFusion: Lidar-Camera Deep Fusion for Multi-Modal 3D Object Detection. Proceedings of the 2022 IEEE/CVF Conference on Computer Vision and Pattern Recognition (CVPR), New Orleans, LA, USA, 18–24 June 2022.

[27] Su S., Delbracio M., Wang J., Sapiro G., Heidrich W., Wang O. (2017). Deep Video Deblurring for Hand-Held Cameras. Proceedings of the 2017 IEEE Conference on Computer Vision and Pattern Recognition (CVPR), Honolulu, HI, USA, 21–26 July 2017.

[28] Cho S.J., Ji S.W., Hong J.P., Jung S.-W., Ko S.-J. (2021). Rethinking Coarse-to-Fine Approach in Single Image Deblurring. Proceedings of the 2021 IEEE/CVF International Conference on Computer Vision (ICCV), Montreal, QC, Canada, 2021.

[29] Sun L., Sakaridis C., Liang J., Jiang Q., Yang K., Sun P., Ye Y., Wang K., Van Gool L. (2022). Event-based fusion for motion deblurring with cross-modal attention. European Conference on Computer Vision.

[30] Kim T., Lee J., Wang L., Yoon K.-J. (2022). Event-guided deblurring of unknown exposure time videos. European Conference on Computer Vision.

[31] Chen L., Chu X., Zhang X., Sun J. (2022). Simple baselines for image restoration. European Conference on Computer Vision.

[32] Sun L., Gehrig D., Sakaridis C., Gehrig M., Liang J., Sun P., Xu Z., Wang K., Van Gool L., Scaramuzza D. (2025). A Unified Framework for Event-Based Frame Interpolation with Ad-Hoc Deblurring in the Wild. IEEE Trans. Pattern Anal. Mach. Intell..

[33] Yang D., Yamac M. (2023). Deformable convolutions and LSTM-based flexible event frame fusion network for motion deblurring. arXiv.

